# Development and explainability of a machine learning prediction model for histological prostatic inflammation in surgically treated patients with benign prostatic hyperplasia: a single-center internal validation study

**DOI:** 10.3389/fmed.2026.1799399

**Published:** 2026-04-21

**Authors:** Chunyang Meng, Lei Peng, Zhiqiang Zeng, Zuoping Wang, Si Ge, Lijian Gan, Yunxiang Li, Jinze Li, Nana Xiang

**Affiliations:** 1 Department of Urology, Beijing Anzhen Nanchong Hospital of Capital Medical University & Nanchong Central Hospital, Nanchong, Sichuan, China; 2 Department of Urology, South China Hospital, Medical School, Shenzhen University, Shenzhen, Guangdong, China; 3 Department of Urology, Deyang People's Hospital, Chengdu University of Traditional Chinese Medicine, Deyang, Sichuan, China

**Keywords:** artificial neural network, benign prostatic hyperplasia, histological prostatic inflammation, machine learning, predictive model

## Abstract

**Background:**

Histological prostatitis is highly prevalent among patients with benign prostatic hyperplasia (BPH) and has been shown to be closely associated with disease progression and postoperative outcomes. However, its diagnosis still mainly relies on postoperative or biopsy pathology, and there is a lack of non-invasive tools to estimate the risk of histological inflammation before surgery or biopsy.

**Methods:**

This was a single-center retrospective machine-learning prediction model study with internal validation, involving 723 patients admitted to the urology department of a tertiary medical center for BPH between June 2020 and June 2023. The cohort was randomly divided into a training set and a validation set in a 7:3 ratio. Least absolute shrinkage and selection operator (LASSO) regression and the Boruta algorithm were used for feature selection, on the basis of which six machine learning models were constructed. Model performance and clinical net benefit were evaluated using the area under the receiver operating characteristic curve, calibration, and decision curve analysis. Shapley additive explanations (SHAP) were applied to provide interpretability at both the global and individual levels, and the best-performing model was further deployed as an online prediction tool.

**Results:**

Among the 723 patients, 387 (53.5%) had histological prostatitis. Of the six machine learning models, the artificial neural network (ANN) model showed the best discriminative ability, with an AUC of 0.852 [95% confidence interval (CI): 0.804–0.899] in the validation set, overall performance superior to the other models, and the lowest Brier score (0.160). It also provided the greatest net benefit across a wide range of threshold probabilities. SHAP analysis indicated that prostate volume (PV), neutrophil-to-lymphocyte ratio (NLR), International Prostate Symptom Score (IPSS), age, systemic immune–inflammation index (SII), and acute urinary retention (AUR) were the key predictors driving model performance; higher values of these variables (or the presence of AUR) were associated with an increased predicted risk of histological prostatitis

**Conclusions:**

Machine learning models, particularly the ANN model, showed good discriminative ability and reasonable calibration for predicting the risk of histological prostatic inflammation. Age, prostate volume, IPSS score, AUR, NLR, and SII were identified as the most important predictors. This prediction model enables accurate and interpretable risk assessment of histological prostatitis in patients with BPH, thereby facilitating early identification of high-risk individuals, supporting refined risk stratification, and optimizing perioperative decision-making.

## Introduction

Benign prostatic hyperplasia (BPH) is one of the most common urological disorders in middle-aged and older men, and its prevalence increases markedly with advancing age (1). BPH is a major cause of lower urinary tract symptoms (LUTS), which can substantially impair quality of life and impose a considerable burden on healthcare resources and socioeconomic systems (2). Accumulating evidence suggests that age-related metabolic disturbances, alterations in sex hormone levels, a variety of inflammation-related cytokines, and chronic prostatic inflammation are all important risk factors for the development and progression of BPH (3, 4). However, the underlying pathogenic mechanisms have not been fully elucidated.

Histological inflammation is frequently observed in prostate specimens obtained from BPH patients undergoing surgery or biopsy (3). Histological prostatitis is increasingly recognized as a potential driving force in the initiation and progression of BPH rather than a mere epiphenomenon (5). Inflammation-related cycles of injury, repair, and regeneration may progressively promote nodular enlargement, accompanied by tissue remodeling characterized by fibromuscular hyperplasia, thereby facilitating the transition of BPH from an asymptomatic stage to clinically overt disease (6). In routine clinical practice, however, prostatic histological inflammation remains a relatively “occult” phenotype, as its diagnosis relies predominantly on invasive sampling, such as prostate biopsy or surgical specimens. Consequently, urologists currently lack reliable non-invasive tools to accurately determine, prior to surgery or biopsy, whether an individual BPH patient harbors significant histological prostatic inflammation. This limitation hinders inflammation-oriented risk stratification and therapeutic decision-making (7).

In addition, systemic inflammation and metabolic dysregulation in patients with BPH have drawn increasing attention. Composite indices derived from complete blood counts, such as the neutrophil-to-lymphocyte ratio (NLR) and systemic immune-inflammation index (SII) are inexpensive and readily accessible surrogate markers of the systemic immune-inflammatory state (8). Recent studies have reported that elevated NLR, SII, and related indices are positively associated with the presence and severity of BPH/LUTS, suggesting that low-grade systemic inflammation may intertwine with prostatic enlargement and symptom burden (9–11). To date, however, there is still a lack of clinically applicable prediction models that integrate these inflammatory markers with conventional clinical characteristics to estimate the risk of histological prostatic inflammation among patients with BPH.

Machine learning (ML) algorithms have been widely applied to disease prediction and risk assessment, providing a feasible approach to constructing predictive models that combine inflammatory biomarkers with clinical features (12). Compared with traditional statistical methods, ML techniques are better suited to handling complex interactions and non-linear relationships, integrating multidimensional data, and enhancing predictive accuracy (13, 14). Nonetheless, many state-of-the-art ML models lack sufficient transparency, interpretability, and comprehensibility, and are often regarded as “black boxes” in clinical settings (15). In recent years, the development of explainable artificial intelligence (XAI) methods has offered new solutions to this challenge (16). Shapley additive explanations (SHAP), a representative XAI approach, can quantify both the direction and magnitude of each variable's contribution to model predictions and present these effects visually, thereby assisting clinicians in understanding the decision-making process and outputs of ML models (17, 18).

The present study aimed to develop and compare multiple ML-based prediction models for histological prostatic inflammation in patients with BPH using routinely available clinical data and inflammatory biomarkers, and to identify the model with the greatest potential for clinical application. By establishing a more accurate risk prediction tool to enable earlier identification of histological prostatic inflammation, this work seeks to facilitate refined risk stratification and individualized management of patients with BPH, ultimately improving their prognosis and quality of life.

## Materials and methods

### Study design and population

**Figure 1:**
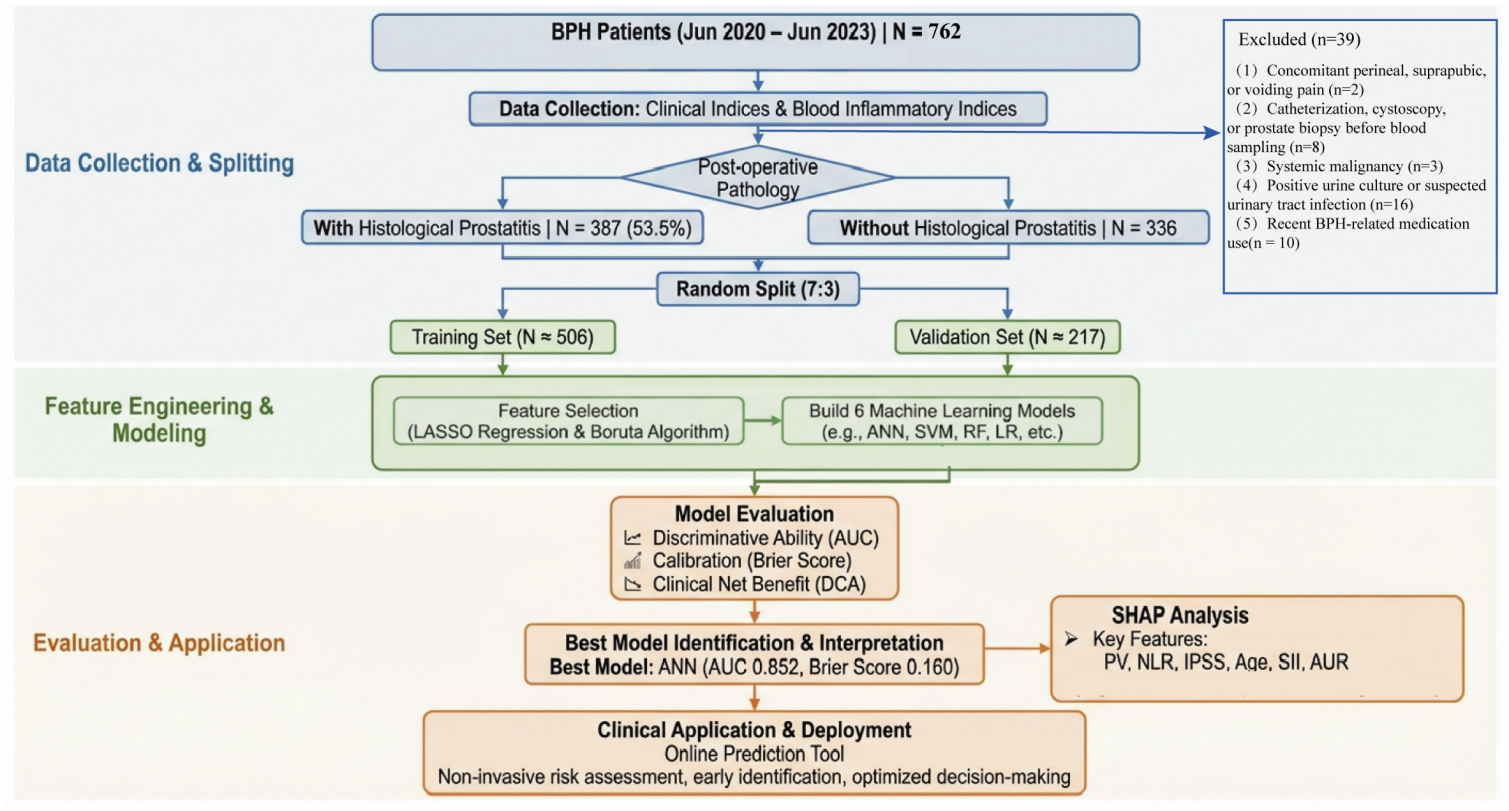
Overall workflow of the study.

This retrospective study was approved by the Ethics Committee of Nanchong Hospital (Nanchong Central Hospital), Beijing Anzhen Hospital Affiliated to Capital Medical University, and written informed consent was obtained from all participants. We adhered to the TRIPOD (Transparent Reporting of a Multivariable Prediction Model for Individual Prognosis or Diagnosis) reporting guideline as well as the STROCSS (Strengthening the Reporting of Cohort Studies in Surgery) guideline (19, 20). The minimum sample size was estimated using the pmsampsize approach for developing a binary prediction model, based on the number of candidate predictors, outcome prevalence, and assumed model performance. The overall study flow is shown in Figure 1.

Clinical data of patients hospitalized with a diagnosis of BPH in the department of urology between June 2020 and June 2023 were collected. Patients were excluded if any of the following criteria were met: (1) clinical presentation of concomitant perineal, suprapubic, or voiding pain; (2) having undergone catheterization, cystoscopy, prostate biopsy, or other urological instrumentation before blood sampling; (3) presence of systemic malignancy; (4) positive urine culture or strong suspicion of urinary tract infection; or (5) use of α1-adrenergic antagonists, cholinergic agents, 5α-reductase inhibitors, or phosphodiesterase type 5 inhibitors within 1 month prior to admission.

### Data collection

All patients completed the Chinese version of the International Prostate Symptom Score (IPSS) and a quality of life (QoL) questionnaire. Demographic characteristics, anthropometric measurements, and laboratory test results were recorded. Body weight (kg) and height (cm) were measured by trained nurses using a standardized protocol. Body mass index (BMI) was calculated as weight divided by height squared (kg/m^2^). Prostate volume (PV) was measured by transrectal ultrasonography and calculated using the ellipsoid formula (height × width × length × π/6). Postvoid residual urine volume (PVR) was also assessed by ultrasound. Maximum urinary flow rate (Qmax) was evaluated by uroflowmetry, and a voided volume >150 ml was required to ensure reliable measurements (21).

At admission, fasting venous blood samples were collected into anticoagulant tubes by professional nurses. Hematological and biochemical parameters were obtained, including neutrophil count, lymphocyte count, platelet count, monocyte count, total cholesterol, triglycerides, high-density lipoprotein, and low-density lipoprotein. The neutrophil-to-lymphocyte ratio (NLR) was calculated as neutrophil count divided by lymphocyte count. The systemic immune–inflammation index (SII) was calculated as platelet count × neutrophil count / lymphocyte count. The prognostic nutritional index (PNI) was calculated as PNI = albumin (g/L) + 5 × lymphocyte count (10^9^/L) (22).

**Figure 2:**
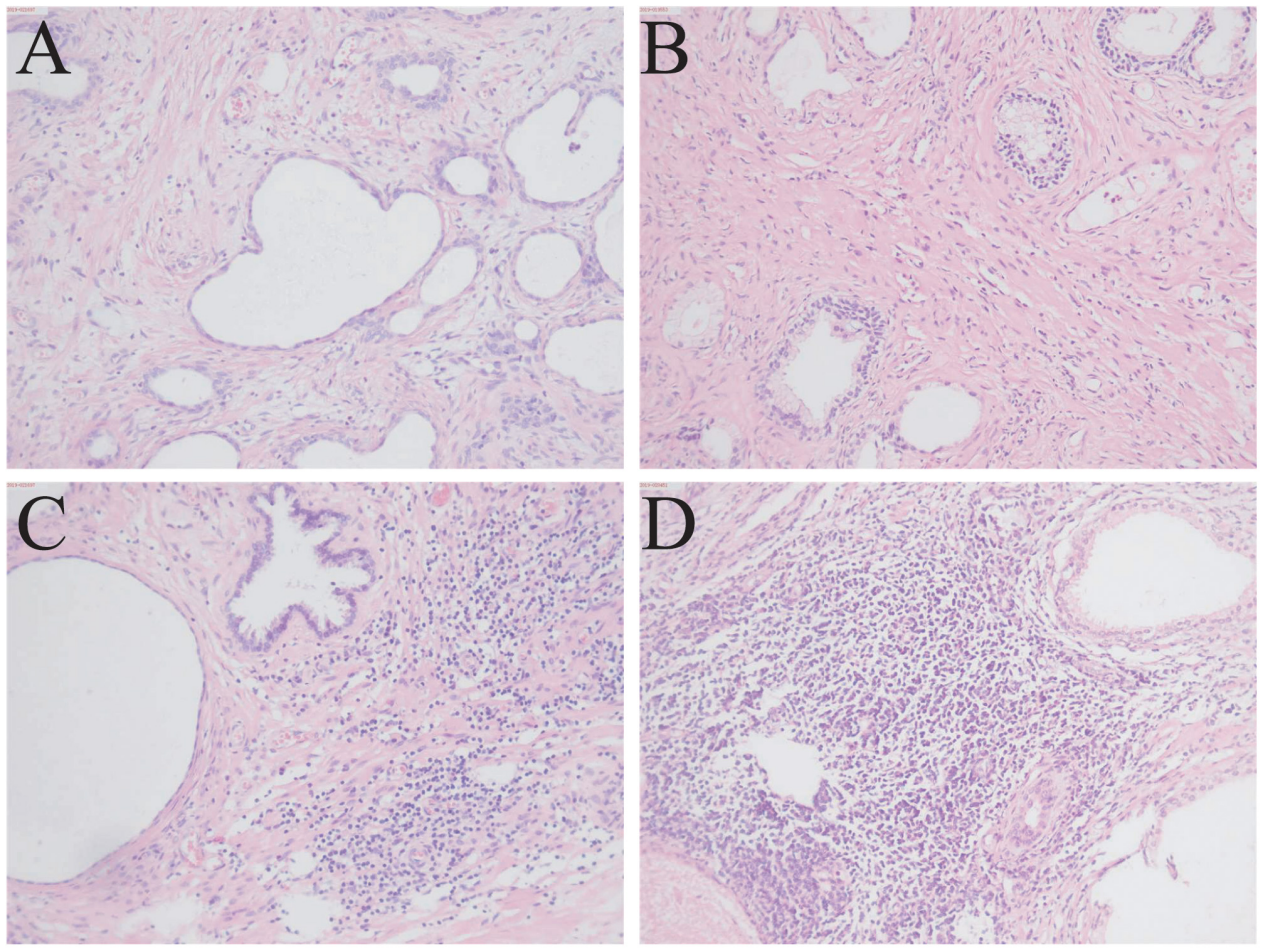
Representative hematoxylin and eosin–stained sections of histological prostatitis(100X). **(A)** No histological prostatitis. **(B)** Mild histological prostatitis. **(C)** Moderate histological prostatitis. **(D)** Severe histological prostatitis.

Postoperative prostate tissue from all patients was independently evaluated by two senior pathologists, and discrepant interpretations were resolved through discussion. According to histopathological classification, prostatic inflammation was graded on a 4-point histological prostatitis scale (grades 0–3) (Figure 2) based on the density and morphological pattern of inflammatory cell infiltration: grade 0, no inflammatory cell infiltration; grade 1, scattered individual inflammatory cells with distinct intervening spaces (< 100 cells/mm^2^); grade 2, confluent sheets of inflammatory cells without tissue destruction or lymphoid nodule/follicle formation (100–500 cells/mm^2^); and grade 3, confluent sheets of inflammatory cells with tissue destruction or nodule/follicle formation (>500 cells/mm^2^). For the main analysis, grades 1–3 were classified as the presence of prostatic inflammation, whereas grade 0 was defined as the absence of inflammation. Detailed criteria are provided in Supplementary Table 1.

### Statistical analysis

The Kolmogorov–Smirnov test was used to assess the normality of continuous variables. Normally distributed data are presented as mean ± standard deviation and were compared between groups using the *t* test. Non-normally distributed data are expressed as median (interquartile range) and were compared using the Mann–Whitney U test. Categorical variables are presented as frequency (percentage) and were compared using the χ^2^ test. To reduce the influence of differences in variable scales and to improve numerical stability and convergence, continuous variables were standardized using z scores before model training. This preprocessing step was particularly relevant for scale-sensitive algorithms such as SVM and ANN (23). Because cases with missing values were excluded prior to modeling, no imputation procedures were applied.

All statistical analyses were performed using SPSS software, with two-sided tests, and a *P* value < 0.05 was considered statistically significant.

### Feature selection

Least absolute shrinkage and selection operator (LASSO) regression was used as a variable selection method suitable for high-dimensional data (24). By imposing an L1 regularization penalty on the regression coefficients, LASSO shrinks some coefficients to zero, thereby achieving feature selection and model simplification. In this study, tenfold cross-validation was applied to determine the optimal penalty parameter λ. The Boruta algorithm, which is based on “shadow features” (i.e., shuffled copies of the original variables), was also used for feature selection (25). In this algorithm, shadow features are introduced into the model together with the original variables; by comparing their importance distributions and accounting for multivariable relationships and interactions, the algorithm identifies and retains only those features with genuine predictive value. The final set of predictors used for model development comprised the common features selected concurrently by both LASSO and Boruta.

### Machine learning algorithms

The dataset was randomly split into a training set and a test set in a 7:3 ratio. The training set was used for model construction, and the test set was reserved for internal validation. Six machine learning algorithms were implemented to build prediction models: decision tree (DT), random forest (RF), extreme gradient boosting (XGBoost), light gradient boosting machine (LightGBM), support vector machine (SVM), and artificial neural network (ANN). In the training set, hyperparameters were tuned using grid search with 5-fold cross-validation. The optimized models were then evaluated in the independent test set for discrimination, calibration, and clinical utility, with discrimination metrics including the AUC, sensitivity, specificity, precision, accuracy, and F1 score (26). Model discrimination was assessed using the area under the receiver operating characteristic curve. The 95% confidence intervals for AUCs were estimated using the DeLong method. Pairwise comparisons of AUCs between models were performed using the DeLong test for correlated ROC curves. In addition, decision curve analysis was performed to assess the clinical net benefit and potential utility of each model across a range of threshold probabilities.

### SHAP

SHAP were used to provide interpretable explanations for the machine learning models and to quantify the impact of each feature on the predicted outcomes. SHAP facilitates interpretation of model predictions at both the cohort and individual levels. For each patient, SHAP values were calculated for all features and then aggregated and averaged to derive global feature importance. SHAP feature importance plots display the overall contribution of each variable, where the bar length corresponds to the mean absolute SHAP value, with larger values indicating greater influence on the model output. SHAP beeswarm plots illustrate the distribution of SHAP values for each feature across all patients; each dot represents an individual's SHAP value for a given feature, with color gradients from red to blue indicating high to low feature values. SHAP dependence plots were generated to characterize the marginal effect of key features on model predictions. In addition, SHAP force plots were constructed to provide local explanations for representative individuals, visually demonstrating how different features collectively shift the model output from the baseline risk toward the final predicted risk for that patient.

All SHAP analyses were conducted in Python using SHAP version 0.40.0.

### Development of the web-based prediction tool

To facilitate clinical application, the final selected machine learning model was deployed as an interactive online prediction tool using the Streamlit framework. By entering the required predictor variables, users can obtain the individualized predicted probability of histological prostatic inflammation in patients with BPH. For practical use, the web-based calculator allows clinicians to enter predictor values in their original clinical units, thereby improving usability despite the standardized representation used during model training.

## Results

### Baseline characteristics of the patients

During the study period, 762 potentially eligible patients were screened. After applying the predefined exclusion criteria, 39 patients were excluded, leaving 723 patients in the final analytical cohort, including 336 patients with BPH without histological prostatic inflammation and 387 patients with BPH with histological prostatic inflammation. The baseline characteristics of the patients are summarized in Table 1. Compared with patients with BPH alone, those with histological prostatic inflammation were older and had a higher incidence of acute urinary retention (AUR), as well as higher IPSS, PV, total prostate-specific antigen (tPSA), aspartate aminotransferase, NLR, and SII. In contrast, patients with histological prostatic inflammation had lower Qmax and high-density lipoprotein cholesterol levels.

**Table 1:** Table1 Clinical characteristics of participants.

	Overall (*n* = 723)	BPH (*n* = 336)	BPH_IN (*n* = 387)	*P* value
Age, median (IQR)	71.46 (67.46, 74.46)	69.46 (65.46, 73.46)	72.46 (68.46, 75.46)	< 0.001
BMI, mean (SD)	24.08 (2.66)	24.12 (2.76)	24.04 (2.57)	0.68
Hypertension, *n*(%)				0.866
Yes	253 (34.99)	116 (34.52)	137 (35.40)	
NO	470 (65.01)	220 (65.48)	250 (64.60)	
Diabetes mellitus, *n*(%)				0.656
Yes	207 (28.63)	93 (27.68)	114 (29.46)	
NO	516 (71.37)	243 (72.32)	273 (70.54)	
Coronary heart disease, *n*(%)				0.183
Yes	159 (21.99)	66 (19.64)	93 (24.03)	
NO	564 (78.01)	270 (80.36)	294 (75.97)	
Smoking.history, *n*(%)				0.332
Yes	383 (52.97)	171 (50.89)	212 (54.78)	
NO	340 (47.03)	165 (49.11)	175 (45.22)	
Alcohol consumption, *n*(%)				0.824
Yes	340 (47.03)	160 (47.62)	180 (46.51)	
NO	383 (52.97)	176 (52.38)	207 (53.49)	
AUR, *n*(%)				< 0.001
Yes	194 (26.83)	57 (16.96)	137 (35.40)	
NO	529 (73.17)	279 (83.04)	250 (64.60)	
Total.cholesterol, mean (SD)	4.91 (0.90)	4.90 (0.94)	4.92 (0.87)	0.795
Triglycerides, median (IQR)	1.42 (0.93, 2.00)	1.38 (0.88, 2.01)	1.43 (0.96, 2.00)	0.176
Total bilirubin, median (IQR)	12.40 (8.60, 16.80)	12.90 (8.40, 17.15)	12.10 (8.95, 16.30)	0.646
HDL-C, mean (SD)	1.14 (0.15)	1.15 (0.14)	1.13 (0.15)	0.045
LDL-C, mean (SD)	2.94 (0.61)	2.94 (0.63)	2.94 (0.60)	0.989
ALT, median (IQR)	22.00 (14.60, 33.35)	21.40 (14.60, 32.97)	22.50 (14.95, 33.40)	0.43
AST, median (IQR)	19.60 (12.90, 29.00)	18.30 (12.17, 28.02)	20.50 (13.65, 30.50)	0.017
ALB, mean (SD)	41.95 (3.91)	41.83 (3.94)	42.05 (3.88)	0.437
GLB, mean (SD)	27.94 (3.89)	28.00 (3.94)	27.90 (3.85)	0.734
A/G ratio, median (IQR)	1.49 (1.33, 1.68)	1.49 (1.33, 1.68)	1.48 (1.34, 1.69)	0.904
IPSS, median (IQR)	18.05 (14.05, 22.05)	15.80 (12.80, 20.80)	19.05 (15.05, 24.05)	< 0.001
QoL, median (IQR)	5.00 (4.00, 5.00)	5.00 (4.00, 5.00)	5.00 (4.00, 5.00)	0.604
Qmax, mean (SD)	7.92 (2.69)	8.15 (2.73)	7.72 (2.63)	0.032
PVR, median (IQR)	61.30 (34.55, 102.20)	65.05 (35.60, 101.58)	58.10 (34.15, 103.50)	0.556
PV, mean (SD)	63.55 (9.18)	59.78 (8.65)	66.82 (8.34)	< 0.001
tPSA, median (IQR)	5.76 (3.63, 7.74)	5.29 (3.34, 7.68)	6.14 (3.98, 7.76)	0.043
fPSA, median (IQR)	0.96 (0.71, 1.18)	0.99 (0.71, 1.20)	0.94 (0.70, 1.16)	0.388
f/t PSA, median (IQR)	0.19 (0.11, 0.31)	0.20 (0.12, 0.30)	0.18 (0.10, 0.31)	0.564
NLR, median (IQR)	2.52 (1.81, 3.52)	2.18 (1.53, 2.76)	2.98 (2.14, 3.92)	< 0.001
SII, median (IQR)	677.20 (571.25, 816.15)	616.35 (537.35, 736.88)	743.70 (618.80, 874.10)	< 0.001
PNI, mean (SD)	52.17 (5.44)	52.19 (5.50)	52.15 (5.40)	0.922

### Feature selection

**Figure 3:**
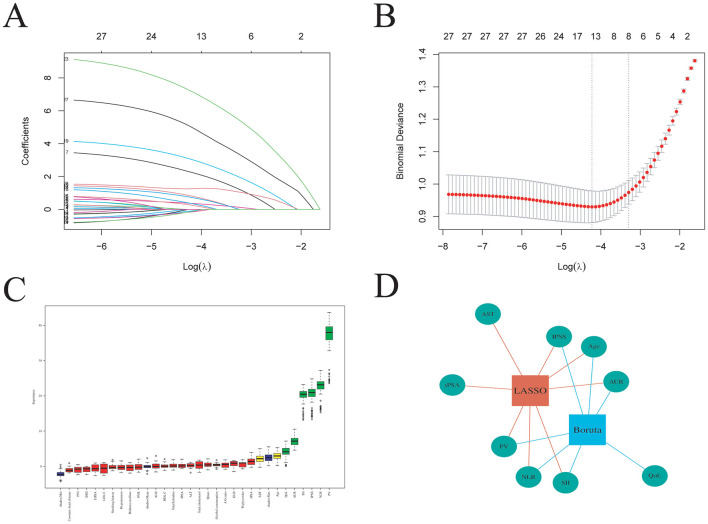
Feature selection using LASSO and Boruta algorithms. **(A)** Coefficient path plot of Lasso regression. **(B)** Ten-fold cross-validation curve for LASSO. **(C)** Feature importance scores ranked by the Boruta algorithm. **(D)** Venn diagram illustrating the overlap of features selected.

To optimize variable selection, both LASSO regression and the Boruta algorithm were applied. After integrating the features identified by these two methods, the final set of predictors used in the machine learning models comprised age, PV, AUR, IPSS, NLR, and SII (Figure 3).

### Comparison of model performance

**Figure 4:**
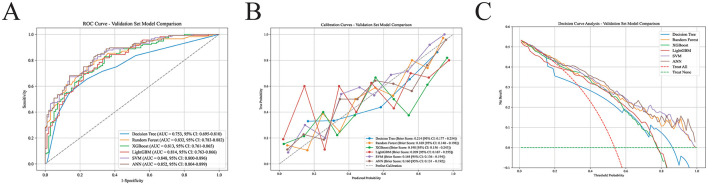
Performance comparison of six machine-learning models in the validation set. **(A)** ROC curves comparing the performance of the six machine-learning models in the validation set. **(B)** Calibration plots of the six machine-learning models in the validation set. **(C)** Decision curve analysis of the six machine-learning models in the validation set.

Based on the selected predictors, six machine learning models were constructed to predict the risk of histological prostatic inflammation in patients with BPH (detailed model parameters are provided in Supplementary Table 2). Receiver operating characteristic (ROC) curve analysis showed that the artificial neural network (ANN) model achieved the best discrimination, with an AUC of 0.852 (95% CI: 0.804–0.899) in the validation set, followed by the SVM model (AUC = 0.848, 95% CI: 0.800–0.896), random forest (AUC = 0.832, 95% CI: 0.783–0.882), LightGBM (AUC = 0.814, 95% CI: 0.763–0.866), XGBoost (AUC = 0.813, 95% CI: 0.761–0.865), and decision tree (AUC = 0.753, 95% CI: 0.695–0.810) (Figure 4A). Pairwise comparisons of correlated ROC curves using the DeLong test showed that the difference in AUC between ANN and SVM was not statistically significant (*P* = 0.269), and the difference between ANN and RF was also not statistically significant (*P* = 0.102). Nevertheless, among the six evaluated models, the ANN model showed the most favorable overall performance profile, with the highest accuracy (0.769), precision (0.775), and F1 score (0.788), as well as the lowest Brier score (Table 2).

**Table 2:** Performance of six models in validation cohorts.

Model	AUC (95% CI)	Accuracy	Precision	Sensitivity	Specificity	F1 Score
Decision tree	0.753 (0.695-0.810)	0.694	0.716	0.716	0.668	0.716
Random forest	0.832 (0.783-0.882)	0.759	0.767	0.793	0.719	0.780
XGBoost	0.813 (0.761-0.865)	0.741	0.721	0.845	0.620	0.778
LightGBM	0.814 (0.763-0.866)	0.736	0.748	0.767	0.700	0.757
SVM	0.848 (0.800-0.896)	0.745	0.790	0.716	0.779	0.751
ANN	0.852 (0.804-0.899)	0.769	0.775	0.802	0.731	0.788

Calibration curves revealed that, for most models, the predicted probabilities aligned reasonably well with the observed event rates, with the calibration curves lying close to the ideal 45° reference line. Notably, the ANN model had the lowest Brier score (0.160), and its calibration curve was closest to the ideal line, indicating superior calibration performance (Figure 4B). Decision curve analysis demonstrated that, across a wide range of threshold probabilities, all machine learning models provided greater net benefit than the “treat-all” and “treat-none” strategies, suggesting potential clinical utility. In particular, the ANN model yielded the highest net benefit across most of the threshold range (Figure 4C).

### Model interpretability

**Figure 5:**
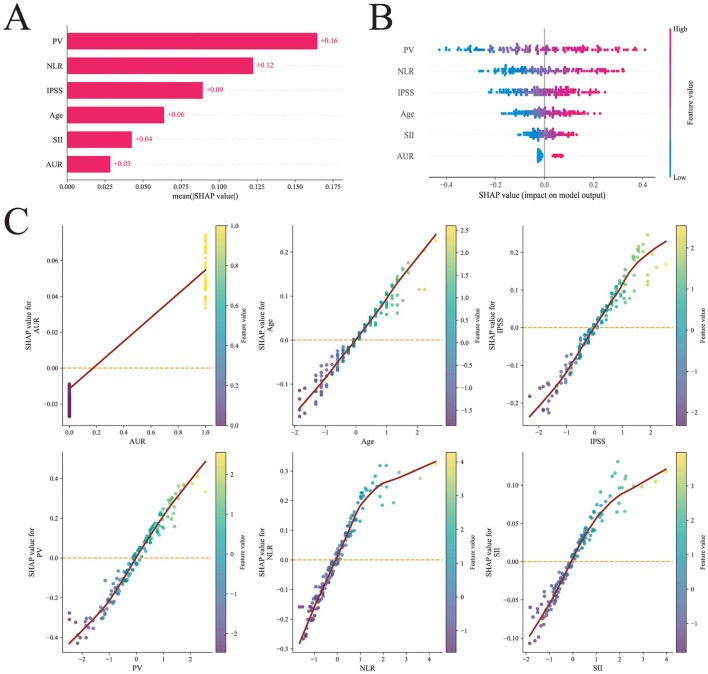
SHAP-based interpretation of the ANN machine-learning model. **(A)** Bar plot of the mean absolute SHAP values showing the relative importance of the six most influential predictors for histological prostatitis. **(B)** SHAP beeswarm plot illustrating the distribution of SHAP values for each predictor; each point represents an individual patient, with color indicating the original feature value (from low to high). **(C)** SHAP dependence plots depicting the marginal effect of each predictor on the model output, showing the relationships between AUR, age, IPSS, PV, NLR, SII and the predicted risk of histological prostatitis.

The SHAP-based feature importance for the ANN model is presented in Figure 5A. Features were ranked in descending order of their mean absolute SHAP values to reflect their relative impact on the model predictions. The most influential predictors were, in order, PV, NLR, IPSS, age, SII, and AUR. The SHAP beeswarm plot illustrates the influence of each feature on the ANN model predictions (Figure 5B). In this prediction model, higher SHAP values for a given feature correspond to an increased likelihood of histological prostatic inflammation. SHAP dependence plots further depict the effects of individual features on the predicted risk (Figure 5C). As PV, age, IPSS, NLR, and SII increased, and as AUR was present, the corresponding SHAP values generally increased, indicating that higher levels (or the occurrence) of these features were associated with an elevated predicted risk of histological prostatic inflammation.

### Patient-level explanation of the machine learning model

**Figure 6:**
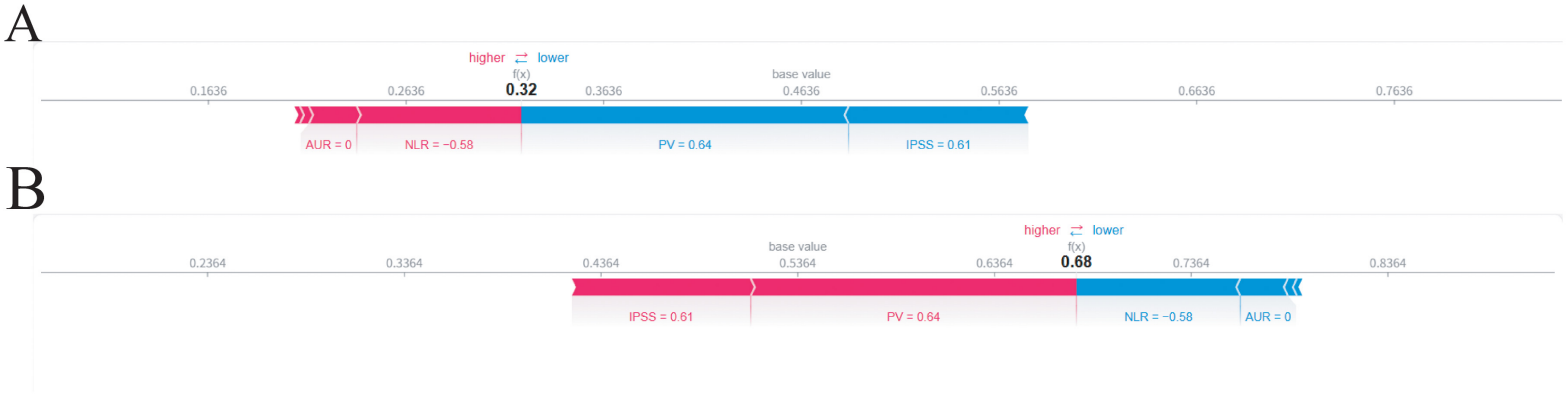
SHAP force plot of a represent patient. **(A)** SHAP force plot for the “no histological prostatitis” class. **(B)** SHAP force plot for the “histological prostatitis” class.

To demonstrate the interpretability of the model at the individual level, a representative patient was selected, and SHAP force plots were generated for the two outcome categories (Figure 6). In these force plots, color indicates the direction of each feature's contribution to the prediction (red for positive, blue for negative), and the length of each bar represents the magnitude of its effect. Starting from the cohort's average prediction (base value), the combination of red and blue feature bars shifts the predicted probability toward the final individualized risk.

For the “no histological prostatic inflammation” category, the base value was approximately 0.46, and the predicted probability for this patient decreased to 0.32 (Figure 6A). For the “histological prostatic inflammation present” category, the base value was approximately 0.54, and the predicted probability increased to 0.68 (Figure 6B). Overall, this pair of force plots shows that, for this patient, higher IPSS and larger prostate volume pushed the prediction toward the presence of inflammation, whereas lower NLR and absence of AUR shifted the prediction toward the absence of inflammation.

### Web-based implementation of the model

To enhance accessibility for clinicians across different centers, the final model was implemented as a user-friendly online prediction tool (https://bph-ann-calculator-zeg6nryionbavhlsy8cmhi.streamlit.app/). By entering the values of the selected predictors, users can obtain an individualized estimated probability of histological prostatic inflammation in patients with BPH. This web tool was developed based on single-center data and should be used with caution until external validation is performed.

## Discussion

The prostate is an immunologically active organ, and its immune responsiveness is closely related to the abundant infiltrates of lymphocytes, macrophages, mast cells, and other inflammatory cells within the gland (4, 27). Histological studies have demonstrated marked aggregation of inflammatory cells in the periglandular region, suggesting that this area may act as a nidus for the initiation and amplification of prostatic immune responses (28). These inflammatory cells and their secreted cytokines can damage epithelial and stromal cells, promote aberrant tissue remodeling characterized by fibromuscular growth, and sustain chronic immune activation (4, 6). In addition, factors such as urinary reflux, metabolic syndrome, alterations in sex hormones, and aging may induce or exacerbate immune dysregulation in the prostate via distinct signaling pathways, thereby establishing a persistent inflammatory microenvironment (29, 30). Increasingly, BPH is being regarded as an immune-inflammatory disease whose onset and progression are, to a considerable extent, driven by chronic prostatic inflammation.

To our knowledge, this is the first study to apply machine learning models to predict histological prostatic inflammation in patients with BPH. We evaluated six different machine learning approaches and found that the ANN model demonstrated the most favorable overall performance, with an AUC of 0.852, an F1 score of 0.726, and a Brier score of 0.160, while also yielding the greatest net benefit across a broad range of threshold probabilities on decision curve analysis. Using SHAP summary and dependence plots, together with individual-level SHAP force plots, we identified age, prostate volume, IPSS score, acute urinary retention, NLR, and SII as key predictors. SHAP-based interpretability further allowed us to explore how these features influenced the predicted risk of histological prostatic inflammation. Finally, we deployed the ANN model as an accessible web-based prediction tool to facilitate clinical implementation.

Histological prostatic inflammation is a common pathological finding in prostate tissue from patients with BPH. Previous studies have reported that the prevalence of BPH with concomitant histological prostatitis varies widely across cohorts (approximately 34.2%−91.7%), largely owing to heterogeneity in sampling strategies and histopathological diagnostic criteria for inflammation (31, 32). In the present study, patients in the histological inflammation group were significantly older than those with BPH alone, suggesting that age-related immunosenescence may contribute to sustained inflammatory infiltration within the prostate and thereby promote the development and progression of BPH (29). Mechanistic and clinical studies have shown that local prostatic inflammation can drive gradual gland enlargement by stimulating stromal cell proliferation, inhibiting epithelial apoptosis, and activating multiple proinflammatory cytokines, with inflammatory burden positively associated with prostate volume and risk of BPH progression (31, 33). Prostatic inflammation is not only linked to bladder outlet obstruction but may also increase the risk of AUR by inducing acute edema and congestion that further narrow the urethral lumen (34, 35). Consistently, patients with BPH and concomitant prostatic inflammation have been reported to exhibit a substantially higher incidence of AUR than those without inflammation (7).

Systemic inflammatory indices derived from complete blood counts have been proposed as surrogate markers for various diseases because they are inexpensive and easily obtainable (7). Prior studies have shown that NLR correlates positively with IPSS, is significantly elevated in patients with moderate-to-severe LUTS/BPH, and is associated with the presence and progression risk of BPH (36). A recent study also found a significant association between NLR and histological prostatic inflammation (11). Moreover, elevated SII has been shown to markedly increase the likelihood of BPH in middle-aged and older adults and to be independently associated with the severity of BPH/LUTS (9, 10). Higher SII levels can not only distinguish controls from patients with BPH but also predict LUTS progression, including an increased risk of AUR and subsequent surgical intervention (37, 38). In our study, NLR and SII ranked among the top features in the SHAP-based importance hierarchy, and higher values of both indices were associated with an increased predicted probability of histological prostatic inflammation.

Surgical studies across different endoscopic techniques have consistently indicated that histological prostatic inflammation is not a trivial incidental finding but rather a high-risk phenotype closely related to perioperative outcomes and postoperative benefit. In a cohort undergoing transurethral bipolar enucleation of the prostate immediately after prostate biopsy, Gu et al. (39) reported that although long-term efficacy was generally comparable, patients with histological inflammation experienced longer operative times, more bleeding- and infection-related complications, and worse early postoperative pain and functional recovery. Similarly, in a prospective study of patients undergoing HoLEP, Zhou et al. found that although LUTS and objective parameters improved significantly in those with inflammation, the magnitude of improvement in IPSS, Qmax, and QoL was substantially less than in patients with BPH alone, suggesting that inflammation is an important predictor of diminished benefit after enucleation (40). Zhang et al. further demonstrated that higher degrees of prostatic tissue inflammation were associated with higher IPSS scores and an increased risk of AUR, and that patients with BPH and histological inflammation had significantly smaller improvements in Qmax and IPSS at 12 months after TUPKRP than those without inflammation (11). However, the diagnosis of histological prostatic inflammation still relies entirely on postoperative or biopsy pathology, which makes it difficult for clinicians to identify, preoperatively, which patients with BPH belong to the “inflammation-driven” high-risk subgroup. Against this background, we sought to integrate routine clinical characteristics and blood-based inflammatory indices to construct a machine learning–based prediction model for histological prostatic inflammation in BPH. Our aim was to enable risk stratification before surgery or biopsy and to provide objective, quantitative support for individualized decisions regarding surgical timing, choice of surgical modality, and more targeted prognostic counseling.

This study has several limitations. First, our machine learning models were derived from a single-center retrospective cohort. Although 5-fold cross-validation and internal validation were performed, inevitable bias remains, and the stability and generalizability of the models still require further validation. Second, the candidate predictors were restricted to routine clinical and hematological variables; data on C-reactive protein, cytokines, metabolic parameters, and imaging features were not available, raising the possibility of unmeasured confounding. Third, we lacked information on sex hormone levels and therefore could not assess their impact on the risk of histological prostatitis. Fourth, the assessment of histological prostatic inflammation was based on pathological sections and may have been influenced by sampling error and interobserver variability. Further prospective, multicenter studies with external validation and incorporation of broader clinical, biochemical, and imaging markers are warranted to refine and validate these prediction models.

## Conclusion

This study demonstrates that machine learning models, particularly the ANN model, showed good discriminative ability and reasonable calibration for predicting the risk of histological prostatic inflammation. Age, prostate volume, IPSS score, acute urinary retention, NLR, and SII were identified as the most critical predictors. Based on these findings, clinicians may more effectively identify high-risk patients at an earlier stage and implement individualized management strategies, thereby promoting more refined risk stratification of BPH and optimizing perioperative decision-making.

## Data Availability

The original contributions presented in the study are included in the article/Supplementary material, further inquiries can be directed to the corresponding authors.
