# Supplementary material for: Development and explainability of a machine learning prediction model for histological prostatic inflammation in surgically treated patients with benign prostatic hyperplasia: a single-center internal validation study
Source: Front Med (Lausanne). 2026 Apr 21;13:1799399. doi: 10.3389/fmed.2026.1799399 (PMC13138966; doi:10.3389/fmed.2026.1799399)
Supplement: Supplementary file 1 [file Table_1.docx]

Supplementary Table 1. Grade of histological inflammation in prostate

| Grade (score) | HP Grade | Description (density of inflammatory cells, number of cells/mm^2^) |
| --- | --- | --- |
| 0 | No HP | No inflammatory cell infiltration |
| 1 | Mild HP | individual inflammatory cells, most of which are separated by distinct intervening spaces (<100) |
| 2 | Moderate HP | confluent sheets of inflammatory cells with no tissue destruction or lymphoid nodule/follicle formation (100-500) |
| 3 | Severe HP | confluent sheets of inflammatory cells with tissue destruction or nodule/follicle formation (>500) |

BPH, benign prostatic hyperplasia; HP, histological prostatitis.
