# Supplementary material for: Development and explainability of a machine learning prediction model for histological prostatic inflammation in surgically treated patients with benign prostatic hyperplasia: a single-center internal validation study
Source: Front Med (Lausanne). 2026 Apr 21;13:1799399. doi: 10.3389/fmed.2026.1799399 (PMC13138966; doi:10.3389/fmed.2026.1799399)
Supplement: Supplementary file 2 [file Table_2.docx]

Supplementary Table 2 Optimal hyperparameter combinations of machine learning models

| Machine learning model | Optimal parameter combination |
| --- | --- |
| Decision tree | {'ccp_alpha': 0.01, 'max_depth': 5, 'max_features': None, 'min_samples_split': 20} |
| Random Forest | n_estimators = 100 , max_features = 2 |
| Extreme Gradient Boosting | {'learning_rate': 0.2, 'max_depth': 3, 'n_estimators': 200, 'subsample': 1.0} |
| Light Gradient Boosting Machine | {'colsample_bytree': 0.6, 'learning_rate': 0.2, 'n_estimators': 100, 'num_leaves': 31, 'subsample': 0.6} |
| Support Vector Machine | {'C': 0.1, 'degree': 2, 'gamma': 0.01, 'kernel': 'rbf'} |
| Artificial Neural Network | {'activation': 'tanh', 'hidden_layer_sizes': (100,)} |
